# BESIDES ACTIVE AGING? A TRANQUIL LIFE AFTER RETIREMENT

**DOI:** 10.1093/geroni/igz038.2752

**Published:** 2019-11-08

**Authors:** Shyhnan Liou

**Affiliations:** National Cheng Kung University, Tainan, Taiwan, Taiwan

## Abstract

This research explores how people think of the time plan after their retirement in rural area in Taiwan. fifty-nine participants in rural communities were interviewed. The findings show patterns of a tranquil life after retirement in three aspects which represent the philosophy mixing in Taiwan (Taoism, Buddhism, and Confucianism). 1). Taking things as they are and following the mandate of heaven(Taoism). 2). the retirement life will not be affected by social changes, and feeling that the pace of life in the society has not changed (Buddhism). 3) When talking about “Future aspirations” and “Future ideal life”, they show concern to their children and posterity. (Confucianism). The findings of tranquil life contribute an alternative way of active ageing with considering the culture. The implications of tranquil life are discussed in the design of elderly education program, technology development to enhance social interaction, and culturally motivated ageing and wisdom.

